# Effective Use of F-18-Fluorodeoxyglucose Positron Emission Tomography/Computed Tomography to Rule Out Prosthetic Aortic Valve as the Source of Infection

**DOI:** 10.7759/cureus.11520

**Published:** 2020-11-17

**Authors:** Pooja Agrawal, James T Roberts, Samuel Bezold, Javier Villanueva-Meyer, Quan D Nguyen

**Affiliations:** 1 Radiology, University of Texas Medical Branch, Galveston, USA

**Keywords:** site of infection, pet scans, ct (computed tomography) imaging, nuclear medicine imaging, aortic valve

## Abstract

Several nuclear imaging techniques can be used to diagnose infectious and inflammatory processes. F-18-fluorodeoxyglucose (FDG) positron emission tomography/computed tomography (PET/CT) is a useful diagnostic technique to detect inflammation and infection quickly and accurately. We report the case of a patient with end-stage renal disease (ESRD) and recurrent bacterial infections where FDG PET/CT was used to identify the source of infection as sternal osteomyelitis and rule out suspected infection of the aortic valve prosthesis.

## Introduction

Infection and inflammation can be detected with various nuclear medicine imaging techniques. For example, imaging with technetium 99m (99mTc) methylene diphosphonate, gallium 67 (67Ga) citrate, indium 111 (111In)-oxine autologous labeled leukocytes, and 99mTc hexylmethylpropylene amineoxime autologous labeled leukocytes can provide diagnostic information; however, each has limitations [1,2]. F-18-fluorodeoxyglucose (FDG) positron emission tomography/computed tomography (PET/CT) is a useful method to detect inflammation and infection quickly and with high sensitivity [3].

FDG PET/CT measures metabolic tissue activity. The uptake of FDG is increased in tissues with increased glucose consumption, such as sites of increased inflammation [4-6]. Inflammatory cells, such as neutrophils and macrophages, have increased glucose transporters [4,6]. Thus, sites of infection can be visualized using FDG PET/CT.

Previous research has found that FDG PET/CT is useful in identifying fevers of undetermined origin (FUO) [7], focal infection [8], and osteomyelitis [9]. Here, we present the case of a patient with suspected infection of a prosthetic aortic valve, and the usefulness of FDG PET/CT in ruling out infection of the valve.

## Case presentation

A man with a past medical history of end-stage renal disease (ESRD) on hemodialysis, aortic dissection, bioprosthetic valve, and recurrent bacteremia was admitted as a transfer for line exchange after he was found to have methicillin-resistant Staphylococcus aureus (MRSA) bacteremia and symptoms of fevers, night sweats, and chills. The patient received vancomycin and subsequent cultures were negative for MRSA, however, repeat blood cultures grew Proteus mirabilis. Multiple sources of recurrent infection were possible, including his permanent dialysis catheter, bioprosthetic aortic valve, or aortic root graft, but the most likely source was thought to be the dialysis catheter. Interventional radiology performed removal of the permanent catheter. Blood cultures and catheter tip cultures were drawn following removal. The catheter tip did not have bacterial growth, however, blood cultures were positive for MRSA and the patient was given appropriate antibiotics.

Given the patient’s history of recurrent infections, the attending team needed to identify the source and discuss its replacement. The attending team discussed whether a gallium scan or tagged WBC scan would be helpful in identifying the source of the infection with the nuclear medicine team. Attending radiologists recommended a PET scan to evaluate prosthesis.

**Figure 1:**
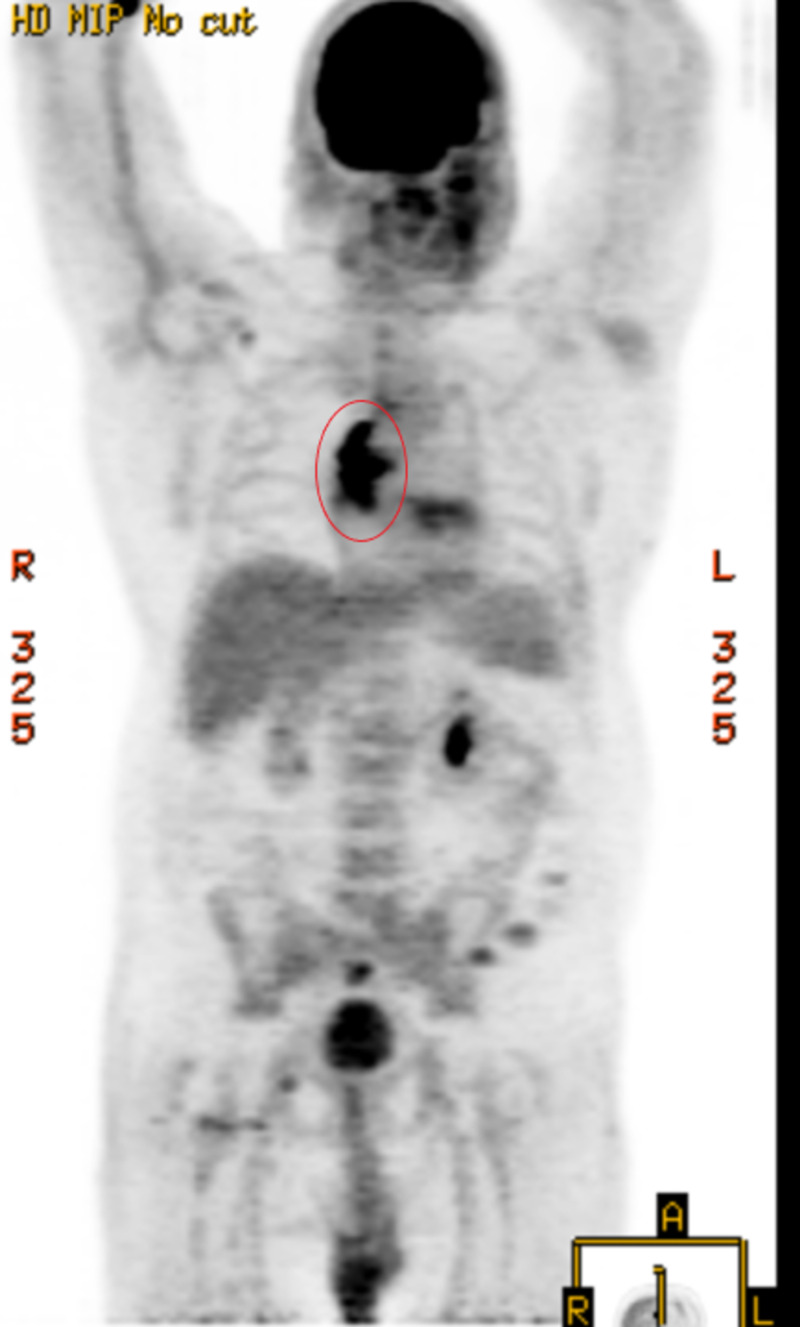
F-18-Fluorodeoxyglucose Positron Emission Tomography Scan A diagnostic FDG PET scan found moderate to marked hypermetabolism within the sternotomy line with maximum standard uptake value (SUV) 4.6. FDG PET: F-18-fluorodeoxyglucose positron emission tomography

**Figure 2:**
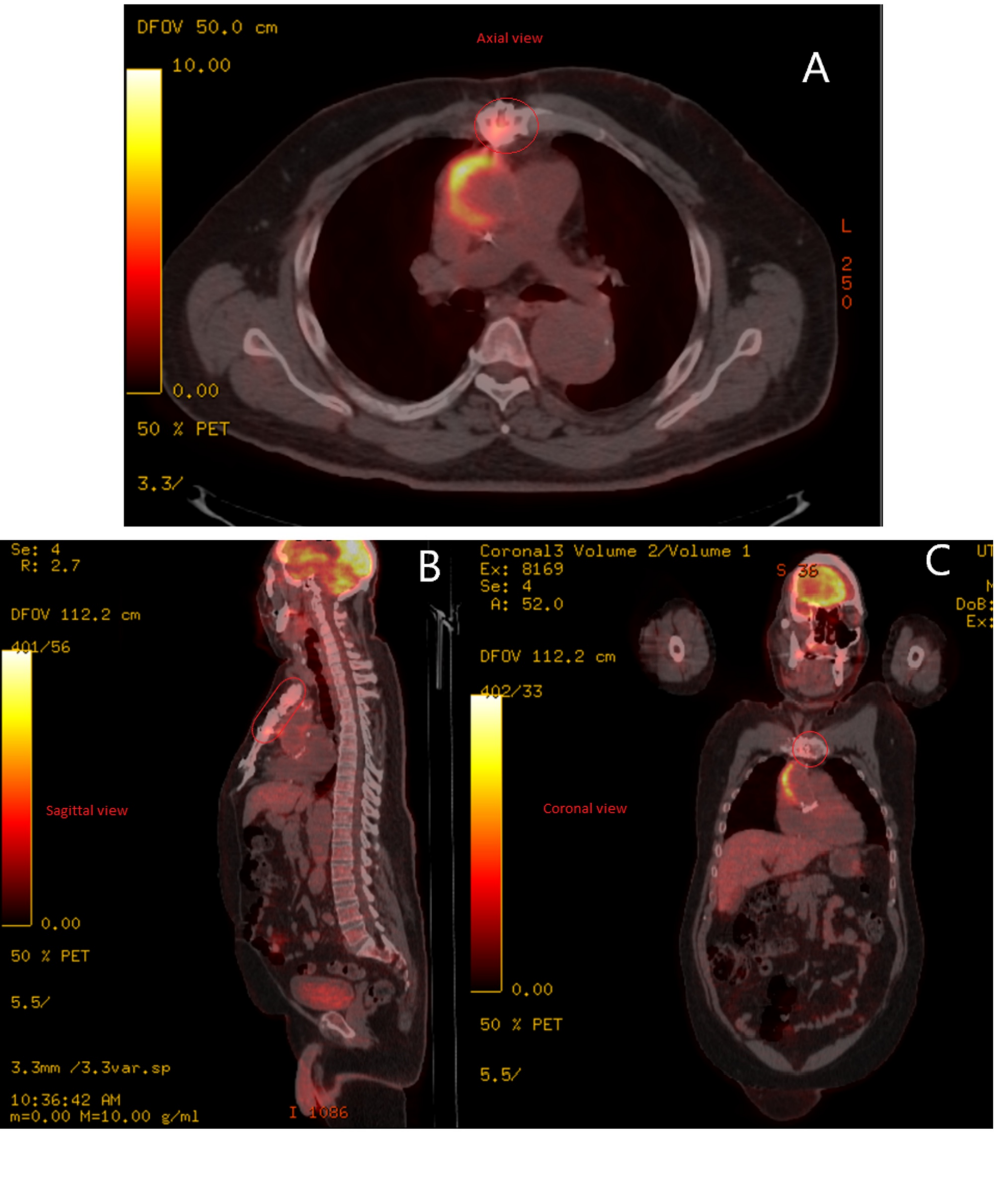
F-18-Fluorodeoxyglucose Positron Emission Tomography/Computed Tomography Scan FDG PET/CT scan revealed hypermetabolism in the upper sternal body with adjacent soft tissue in the anterior mediastinum, suspicious for osteomyelitis. An additional crescentic collection was found along the right aspect of the ascending aortic arch that did not extend along with the prosthetic valve. The scan includes axial (A), sagittal (B), and coronal (C) views. The red circles denote sites of increased FDG uptake. FDG PET/CT: F-18-fluorodeoxyglucose positron emission tomography/computed tomography

The scan revealed hypermetabolism in the upper sternal body, suspicious for osteomyelitis (Figures 1, 2). The bone in the region of hypermetabolism was abnormal. The sternotomy was unfused and showed no signs of healing. The margins of the upper sternotomy, which is the region of osseous hypermetabolism, were irregular with areas of erosion. More inferiorly, the sternotomy showed callus formation, no erosions, and no hypermetabolism.

An additional crescentic collection was found along the right aspect of the ascending aortic arch, however, it did not extend along with the valve. The collection was most likely a hematoma, measuring above fluid attenuation by CT. Whether there was infection superimposed upon the hematoma is unknown, though possible. While the collection abutted the prosthetic valve, there was no other uptake around the valve to suggest infection of the prosthesis. Thus, the source of infection was likely from the sternum and aortic arch and not from the aortic valve prosthesis.

The patient’s antibiotic therapy was extended. A second PET scan confirmed the infection was not from the aortic valve prosthesis. The patient underwent AV fistula creation for permanent dialysis access.

## Discussion

A variety of nuclear imaging studies can aid in the diagnosis of infection, particularly in complex cases of recurrent bacteremia, such as the case presented above. MRI is used often in infection imaging but has prolonged acquisition times and may have inconclusive results in certain infectious processes [10,11]. White blood cell imaging is another technique that may be used, but it exhibits a low range of sensitivity in chronic infection, such as chronic osteomyelitis [10]. FDG PET/CT is a particularly useful diagnostic technique to identify the source of infection. FDG PET/CT also shows increased uptake in bone lesions, such as brown tumors. Advantages include short acquisition time, high-resolution, and increased sensitivity in chronic and spinal infection [10].

FDG PET has been shown to have value in the diagnosis of chronic limb osteomyelitis, where it has high rates of specificity and sensitivity [12,13]. For example, the sensitivity and specificity of FDG PET were found to have sensitivity and specificity over 95% in the diagnosis of osteomyelitis [13]. Three-phase bone scintigraphy and labeled white cell scintigraphy have comparatively lower rates of sensitivity and specificity [12].

The infection of a prosthetic aortic valve is associated with high rates of morbidity and mortality [14]. This is partly due to a delay in diagnosing infection and identifying the source [15]. Patients may also present with a wide range of symptoms, delaying accurate diagnosis and treatment [15]. Thus, it is imperative to quickly identify the source of infection to result in improved outcomes. Further, if the infection of a prosthetic aortic valve is suspected, accurate diagnosis is vital, given the high rates of mortality associated with valve replacement [16]. In this setting of a patient with recurrent bacteremia, FDG PET/CT was helpful in ruling out infection of aortic valve prosthesis (and thus avoiding high-risk surgery) and diagnosing osteomyelitis.

## Conclusions

FDG PET/CT can be used to identify the source of infection or exclude certain infectious processes with high sensitivity. FDG PET/CT ruled out infection of the aortic valve prosthesis in this patient with recurrent bacteremia. Given its increasing availability, as well as rapidness and relatively lower cost compared to labeled WBC scan, FDG PET/CT should be considered in the diagnosis of infectious processes.
